# Proof-of-PUF Enabled Blockchain: Concurrent Data and Device Security for Internet-of-Energy

**DOI:** 10.3390/s21010028

**Published:** 2020-12-23

**Authors:** Rameez Asif, Kinan Ghanem, James Irvine

**Affiliations:** 1 Power Networks Demonstration Centre (PNDC), University of Strathclyde, Glasgow G1 1XQ, UK; kinan.ghanem@strath.ac.uk; 2 Department of Electronics and Electrical Engineering, University of Strathclyde, Glasgow G1 1XQ, UK; j.m.irvine@strath.ac.uk

**Keywords:** internet-of-energy, smart grid, blockchain, artificial intelligence, cybersecurity, cyber physical systems, data processing, encryption, cloud computing, communication systems

## Abstract

A detailed review on the technological aspects of Blockchain and Physical Unclonable Functions (PUFs) is presented in this article. It stipulates an emerging concept of Blockchain that integrates hardware security primitives via PUFs to solve bandwidth, integration, scalability, latency, and energy requirements for the Internet-of-Energy (IoE) systems. This hybrid approach, hereinafter termed as PUFChain, provides device and data provenance which records data origins, history of data generation and processing, and clone-proof device identification and authentication, thus possible to track the sources and reasons of any cyber attack. In addition to this, we review the key areas of design, development, and implementation, which will give us the insight on seamless integration with legacy IoE systems, reliability, cyber resilience, and future research challenges.

## 1. Introduction

**Figure 1:**
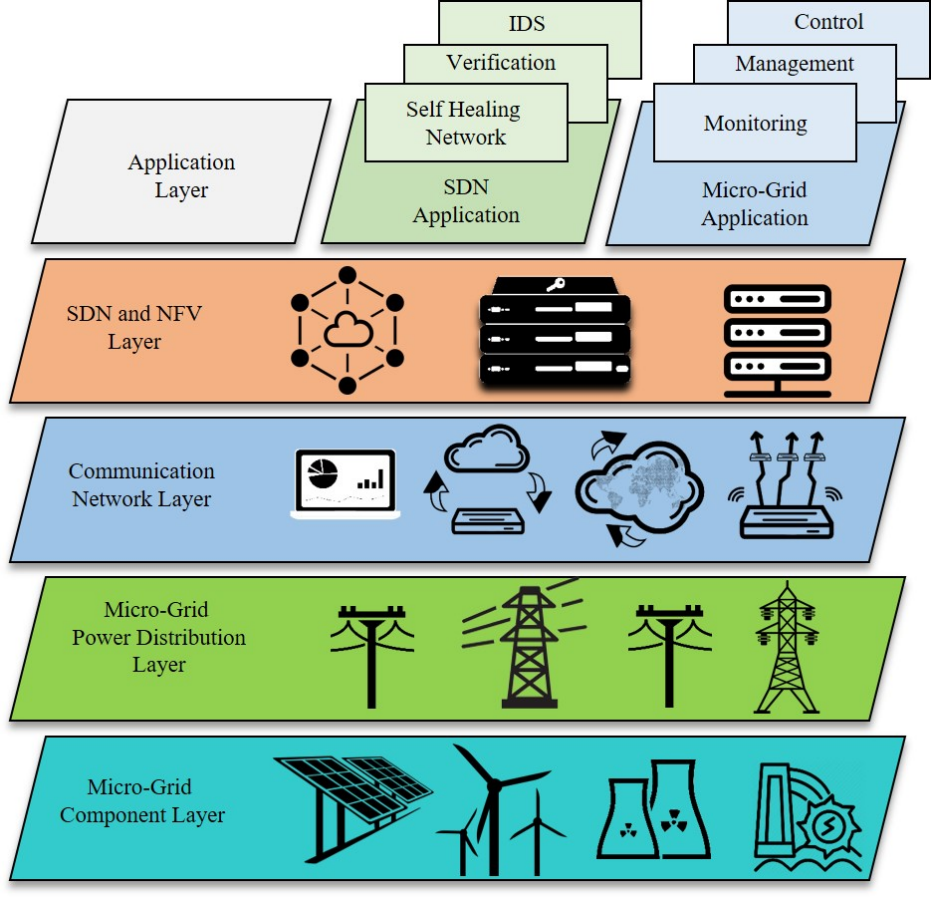
Basic IoE Framework. [Integration of information and communications technology (ICT), cybersecurity, encryption, cloud computing, and energy systems to form Internet-of-Energy (IoE)].

The expeditious development in the areas of information and communications technology (ICT), cybersecurity, encryption, cloud computing, as well as energy systems has led to the emergence of a new concept called as Internet-of-energy (IoE) [1]. The IoE is the result of the implementation of Internet-of-Things (IoT) technology [2], smart senors [3,4], and intelligent data analytics [5] with distributed energy systems. The foremost purpose is to maximise the efficiency of the generation, transmission, digitisation, and utilisation of electricity [6]. IoT technology enables the IoE by creating networks of smart sensors that have numerous smart grid applications [7]. These include power monitoring, demand-side energy management, secure OT/IT convergence, distributed storage, and renewable energy integration among others as depicted in Figure 1.

Distribution Network Operators (DNOs) continue to move into a future, where IoE [8,9,10] is driven by the combination of secure intelligent mobile-edge devices and high-bandwidth communications with real-time data analytics [11]. These next-generation technologies lead towards virtualisation [12,13], security [14,15,16], automation [17,18], and digitalisation [19] capabilities that enable the use and management of more sustainable resources in operational technology (OT). However, the utility sector still faces the challenge of integrating the data and software subsystems [20] required with using complex security protocols [21] and communication architectures [22,23] to unlock and enable the desired functionalities. The quintessential solution for this challenge lies in the security framework, virtualisation, intelligent cloud-based platforms for computational efficiency, data storage, and implementing codeveloped cloud-based grid applications [24]. The cost of industrially focused resilient and robust computing hardware can be substantial and dependent on the number of edge devices required per substation [25] and the level of redundancy required. For providing seamless hardware integration and cost effective solutions to the substation automation, the use of identification, verification, and virtualisation can be deployed to provide cyber-secure remote access [26].

When edge devices are implemented in IoE networks, they are vulnerable to external cyber threats and are an easy target [27,28]. Not only are these cyber threats restricted to the hacking of data contained on the computer itself, they may also use the trustworthy device status to obtain access to other interconnected networks [29]. During the deployment process or during the firmware upgrade, malicious hackers may take advantage of the opportunities to inject unauthorised code to get access to the processing hardware. To create counterfeits and clones, sophisticated cyber criminals or insider attackers with privileged access to the devices may take advantage of unsecured manufacturing systems, resulting in significant financial, environmental, and infrastructure disruption [30]. The security frameworks and protocols are vital at remote or third-party facilities, where the devices and the data-management is centrally connected via a control room [31]. Over the last few years, there has been a tremendous interest in developing fit-for-purpose algorithms to include IoT data and system level security/privacy [32,33,34,35,36]. IoT systems typically accumulate big data sets, some of which require encryption depending on criteria for sensitivity or compliance [37]. IoT data protection solutions must be an end-to-end solution, i.e., span between edge to the cloud, provide scalable encryption and key management [38]. We have summarised the cyber attacks in the IoE environment in Table 1. In general, the most effective way to protect IoE devices in a fragile setting is currently by cryptographic methods. However, when applying these cryptographic approaches in IoE networks, power consumption and key storage are among the major concerns.

Blockchain has received world wide attention among the industry, the Government, and academia alike to dispense security and privacy to the assets. Blockchain, sometimes referred to as Distributed Ledger Technology (DLT), makes the transaction records of any digital asset unalterable and transparent via decentralisation and cryptographic hashing [39]. Despite using Blockchain in the IoT networks [40,41], there should be protection mechanisms in place to verify and authenticate the edge devices through hybrid technologies such as Blockchain in combination with Physical Unclonable Functions (PUFs) [42]. PUFs evaluate manufacturing variations in integrated circuits (ICs) and generate an individual response for each device, also known as fingerprint [43]. This response significantly varies from chip to chip and can be used for identification and authentication purpose.

**Table 1:** Taxonomy of attacks based on different layers of the TCP/IP model in IoE domain [44].

TCP/IP Layer	Attack	Attack Vector
Physical	Jamming [45]	With radio interference
	Tampering [46]	Making fake nodes
Data Link	Collision [47]	Transmit data at the same time in the same frequency channel
	Exhaustion [48]	Multiple collisions and continuous re-transmission until the node runs out of resource
	Unfairness [49]	Repeatedly ask for the channel to limit others request
Network	Spoofed, or Replayed routing information [49,50]	Routing loops, changing the source of the route, Repelling network from selected nodes
	Selective forwarding [51]	Send selected information to the legitimate receiver
	Sinkhole [52]	Become the target of all nodes in order to gather all information
	Sybil [53]	Create lots of pseudonymous identities to undermine the authorised system
	Wormholes [54]	Re-transmit information to the IoT nodes
	Hello flood [55]	Use Hello messages to flood the network with these tiny messages
	Acknowledgement spoofing [56]	Spoof the link layer acknowledgement
Transport	SYN flooding [57]	Resend request multiple times to fill the capacity of the transport layer
	Desynchronisation [58]	Reinitialise the connection in order to disrupt it
Application	Reliability attacks: Clock skewing, Selective message Forwarding, Data aggregation distortion [59,60]	Impersonate itself as a reliable node in the IoT network and sends corrupted data

In this article, we have reviewed the hybrid use of PUFs and Blockchain for IC traceability, verification and authentication in IoE systems. The consensus framework, based on Proof-of-PUF (PoP), aims to guarantee authentication of the devices and the miner with a rapid verification process compared to existing Blockchain consensus algorithms. The combination of Blockchain and PUF allows us to propose an efficient framework that guarantees data provenance and device integrity in IoT networks. This review contributes to: (a) an in-depth market survey of the IoE systems and energy utilities, (b) current research and development work on Blockchain and PUFs, their implementation methods and algorithms, (c) cross-disciplinary study of PUFChain approach that can simultaneously handle device and data security, (d) evaluation of the cyber resilience of the PUFChain, (e) applications and use-case study of PUFChain and (f) research challenges, issues, and future developments.

## 2. Market Survey of IoE

The global IoE market has been segmented by hardware and software based real-time data analytics [61], security frameworks, data storage management, remote monitoring, and others [62]. Among these categories, remote monitoring software in the energy sector is projected to expand over the forecast period at a substantial compound annual growth rate (CAGR). This can be due to the growing need for remote monitoring systems with devices activated by the internet. Energy management also makes it possible for different end-use industries such as electricity, oil, and gas and mining industries to reduce energy shortages, further reducing running costs [63]. One of the key reasons driving the demand for Internet-of-Things (IoT) devices and applications is the rising concern about energy management.

In 2015, the global IoE market hit USD 6.8 billion and is forecast to hit USD 26.5 billion by 2023, with a CAGR of 15.5% percent over the 2016–2023 period. Factors such as increasing globalisation combined with urbanisation are projected by the end of the forecast period to broaden the IoE market by notable sales. IoE’s large market share applies to inter- and intraconnected IoT devices. The number of devices connected to the internet now exceeds the number of people on the planet. According to analyst firm Gartner, (Gartner, Newsletter August, 2019) there are 8.4 billion devices—or ”things”—connected to the Internet in 2017. By 2020 it predicts the number of IoT devices will rise to 20.4 billion worldwide. Furthermore, this growth is not only in the home, with devices such as smart TVs, smart fridges, and security cameras but also massively at the industrial level. Increasingly businesses are connecting devices to the internet and turning them into intelligent assets that can help drive greater efficiencies, increase competitiveness, develop new business models, and provide solutions to problems. By 2023 new low-power Internet-of-Things technologies such as lower-power wide-area network (LP-WAN) will help drive the next wave of IoT adoption, with its promise of increased secure network coverage and cost effective seamless integration. Furthermore, IoT can help businesses take up other technologies such as cloud services, end-to-end lightweight encryption, Blockchain, artificial intelligence (AI), and machine learning (ML).

Energy sector will be the highest consumer of IoT edge devices [62], totalling 1.17 billion devices in 2019 and increasing 17% in 2020 to reach 1.37 billion devices. In 2020, domestic energy smart metering, which can be used for more intelligent metering and forecasting, will be the top usage case for Greater China and Western Europe, comprising 26% and 12% of total IoT devices, respectively, as compared to 2019 (Global Market Insights, IoT Utilities Market Size, Growth—Industry Share Forecast Report 2024). In contrast, North America will see the largest acceptance of IoT endpoints in the electricity, renewables, and asset security industries, comprising 8% of the overall IoT devices. Despite the progress made on standards and technologies, i.e., edge computing, LPWAN, cloud integration, bandwidth allocations, private mobile connectivity and data analytics, IoT ecosystems remain complex because of the unavailability of the unified standards, lightweight security/communication protocols, and a unified firmware platform that can provide the orchestration between the multivendor equipment [64]. The current fragmentation in IoT connectivity and technical solutions has impacted equipment costs and reduced interoperability. The latest market survey (Ofcom, Data Assets, Final Report, April 2014) focused on 12 key growth areas in IoT and found the complex nature of IoT ecosystems but massive increase in the number of connections. The number of IoT connections in the identified sectors are predicted to grow from around 13 million in 2016 to over 150 million by 2024 in the UK.

**Figure 2:**
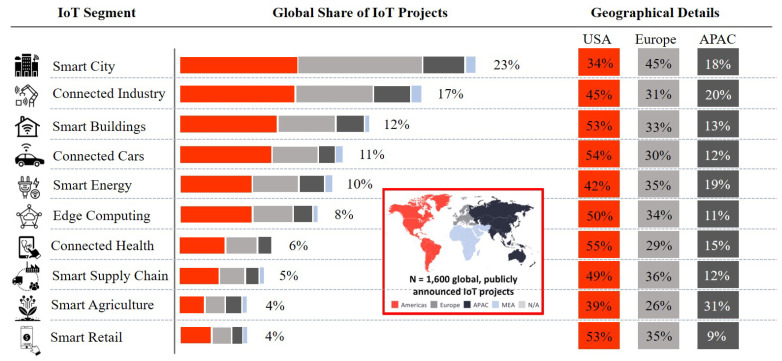
Statistics of Internet-of-Things (IoT) segments according to 2018 project and market survey with increasing trends in smart energy, edge computing, and smart supply chain. (Data Source: IoT-Analytics, Janury 2018. Based on 1600 enterprise projects announced world wide and not including consumer wearable IoT projects).

The key variables driving the demand for IoT and intelligent edge computing, as in Figure 2, growing industry-wide acceptance of IoT connectivity; increasing demand for low-latency and real-time, automatic decision-making solutions; and the need to overcome the exponentially increasing volume of data and network traffic [65]. Countries around the world are introducing edge computing technologies to increase the efficiency of the energy market, EV infrastructure, building safety, home automation, parking and traffic management, and community resources management with the growing drive into smart city initiatives. Edge computing empowers IoT deployments in smart cities with low-latency and high bandwidth connectivity together with cloud integration and data analytics as a service. The advantage of this modern matrixed thought on the optimisation of intelligent edges is that it:

- Improves responsiveness by reducing decision-making latency
- Increases data security and privacy
- Requires less power
- Uses less network bandwidth
- Maximises efficiencies, reliability, and autonomy
- Reduces infrastructure and operational costs

It is also worth mentioning that 7% of all world-wide IoT projects incorporated the new generation and upcoming Low-Power Wide-Area (LPWA) connectivity technology [66]. Approximately 64% of the projects are focusing on smart city initiatives, smart energy infrastructures, mobile edge computing and industry 4.0. LoRa technology shared 37% of these projects followed by SigFox (21%) and NB-IoT (19%) (IoT Analytics, Projects Data, Jan 2018). The main value driver for most IoT enterprise projects is cost savings (54%). Only 35% of IoT projects are used to increase per annum capital (e.g., by offering new IoT-connected products and services). Twenty-four percent of projects also increase overall safety, i.e., by offering enhanced telemonitoring systems with real-time emergency notifications, authentication, and verification [67].

## 3. Distributed Ledger Technology (DLT) and Blockchain

**Figure 3:**
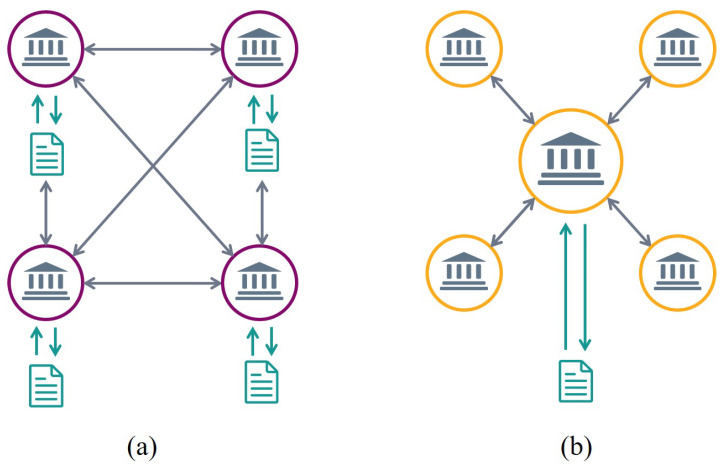
Implementation of; (**a**) Distributed Ledger Technology (DLT) and (**b**) Blockchain (Centralised Ledger).

DLT is moving quickly from theory to practical applications, transforming the data management and exchange in a secure manner. Unlike centralised databases, DLT stores information on synchronised independent processors, controlled by the individual participants in the network, which increases security, privacy, and interoperability [68]. DLT is an umbrella technology with benefits such as decentralisation, persistence, anonymity and audit-ability [69]. While Blockchain is termed as the first fully functional implementation of DLT, as shown in Figure 3. There is a wide spectrum of Blockchain applications ranging from 5G/4G-LTE communications, energy utilities, health care, smart cities, crypto-currency, financial services, risk management, Internet-of-Things (IoT) to public and social services [70].

**Figure 4:**
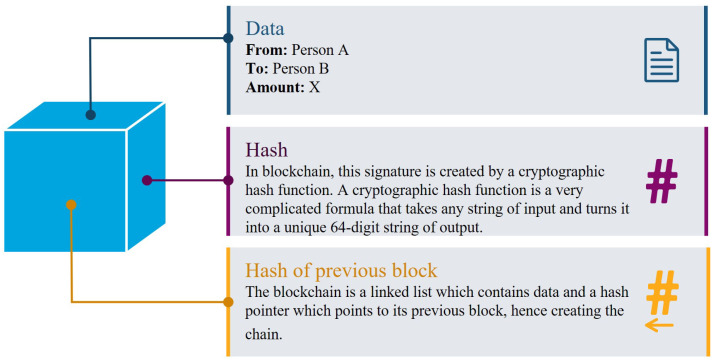
The basic block architecture of Blockchain and information encoded in each step.

More specifically, Blockchain technology incorporates a decentralised ledger that operates in a transparent environment. Each block of the ledger contains data about transactions that have been executed on the platform. The blocks are formulated by the data information, hash functions and hash of the previous blocks, as depicted in Figure 4, hash is a mathematical computation that fulfils the encrypted demands required to solve for a Blockchain computation. A hash, like a nonce or a solution, is considered as the backbone of the Blockchain network [71]. Hashing requires processing the data from a block through a mathematical function, which results in an output of a fixed length. Using a fixed-length output increases security [72] since anyone trying to decode the hash won’t be able to tell how long or short the input is, as they are one-way functions. In order to add a block to the ledger, every edge node of the IoE network needs to be verified and identified. Thanks to this verification the system does not need an intermediary to check transactions. Information stored in a Blockchain can never be deleted and serves as a verifiable and accurate ledger of every transaction made within the system [73,74].

The benefit of using Blockchain algorithms in the IoE networks [75,76,77,78,79,80] is twofold. It can not only provide data security and privacy but also can be used for the mobile edge devices verification and identification [81,82]. For energy utilities, large amounts of data are gathered from edge terminals or Internet-of-Things (IoT) devices; however, data trust and security in edge computing environment are very important issues to be considered for future OT/IT converged solutions, especially when the gathered data are fraud or dishonest, or the data are misused or spread without any authorisation, which may lead to serious cybersecurity problems. Blockchain can also facilitate the development of the Digital Twins (DTs) in IoE, to guarantee secure, private and trusted traceability, accessibility, and immutability of records, logs, and data provenance [83]. Beside the inherited capabilities of Blockchain to provide security and privacy, the orchestrated Blockchain networks [84] decrease system drop rate and further offload the cloud data-centre with seamless integration in existing IoE ecosystem. The reduced computational workload from cloud data-centre does not only help in saving the CAPEX and OPEX; it is beneficial for the energy constrained devices and minimising carbon emissions in environment.

### 3.1. Blockchain Networks

The functionality and the performance of the Blockchain depends on the architecture of deployed network. Based on the implementation, the Blockchain networks are broadly categorised as Public [81] and Enterprise networks [85]. The pros and cons of the two network categories are discussed in this section.

#### 3.1.1. Public Blockchain (Permission-Less Blockchain)

A public Blockchain network is an architecture that can be read, sent records and expected to be included by everyone in the world provided they are checked as a node and anyone can take part in the consensus process [86]. The fusion of economic rewards and cryptographic authentication using methods such as proof-of-work (Bitcoin) or proof-of-stake (Ethereum) is primarily covered by cryptoeconomics. These Blockchains are generally considered to be “fully decentralized” [87]. One of the disadvantages is the considerable amount of computing power (resources) needed to sustain a large-scale distributed ledger, especially in the densely deployed IoE network [88]. Shared Blockchain networks have a means of shielding the end-user from the developers, establishing that there are stringent requirements in place.

#### 3.1.2. Enterprise Blockchain (Permissioned Blockchain)

Enterprise networks are often referred to as networks of private entities or consortium. An enterprise is a Blockchain where consensus is managed by a number of preselected nodes [89]. The consortium comes together and make decisions for the whole network. Hence, it is termed as consortium Blockchain or federation Blockchain. In a completely private Blockchain, the only drawback being that rights are held by one entity. The Blockchain enterprise decreases processing expenses and data redundancies, removes obsolete processes, simplifies record management, and avoids semimanual enforcement frameworks [90]. This network architecture for densely deployed IoE networks is strongly recommended. Since read permissions are restricted, enterprise Blockchains offer a higher degree of privacy and protection [91].

### 3.2. Consensus Algorithms in Blockchain

A consensus algorithm is a process that allows users or machines to coordinate in a distributed network [92]. These algorithms make sure that all nodes in the system can agree on a single source of truth (verification or proofs), even if some nodes fail [93]. The need for a single source of truth originated the notion of replicated database systems in order to ensure resilience against node failures within an architecture. Such database systems ensure that data is not lost when one or more nodes fail to function in an excepted fashion [94]. In this section, we explore different incentivised consensus algorithms. Such algorithms can be grouped in two major categories: Proof-of-Work (PoW) and Proof-of-Stake (PoS) [95].

#### 3.2.1. Proof-of-Work (PoW)

In PoW algorithm, the probability of mining a block is determined by how much computational work is done by the miner (verified node). A PoW mechanism involves two different parties (nodes): prover (requestor) and verifier (provider). The prover performs a resource-intensive computational task intending to achieve a goal and presents it to a verifier or a set of verifiers for validation that requires significantly less resource [96]. In PoW, the framework sets out conditions for what makes a block valid and verified. It might say, for instance, only a block whose hash begins with 00 will be valid. The only way for the miner to create one that matches that combination is to brute-forcing inputs. They can iterate a parameters in the data-pool to produce a different outcome for every guess until they get the right hash. With major Blockchains, it is cumbersome to compete with other miners, as high performance computing and special hashing hardware (ASICs) [97] is required to achieve the required validation of the block.

#### 3.2.2. Proof-of-Stake (PoS)

In PoS algorithm, the probability of validating a new block is determined by how large of a stake an individual possesses [98]. The core idea of PoS evolves around the concept that the nodes who would like to participate in the Blockchain architecture must prove that they own a certain number of coins at first [99] that can be translated as credibility and experience. Besides, they must lock a certain amount of their stakes, into an escrow account [100,101] in order to participate in the block creation process. The stake acts as a guarantee that it will behave as per the protocol rules.

In recent years, there has been a lot of interest in developing consensus algorithms, they are summarised in Table 2.

**Table 2:** Summarised Consensus Algorithms for Blockchain.

Property	PoW [96]	PoS [98]	PBFT [102]	DPoS [103]	Ripple [104]	Tendermint [105]
Node Identity Management	Open	Open	Permissioned	Open	Open	Permissioned
Energy Savings	No	Partial	Yes	Partial	Yes	Yes
Tolerated Power of Adversary	<25% Computing Power	<51% Stake	<33% Faulty Replicas	<51% Validators	<20% Faulty Nodes	<33% Byzantine Voting
Transactions (per second)	7–30	30–173	100–2500	25–2500	500–1500	10,000
Transactions Fee	High	Low	Very Low	Low	Very Low	High
Use-Cases	Bitcoin	Peercoin	Hyperledger Fabric	Bitshares	Ripple	Tendermint

### 3.3. Interoperable DLT and Blockchain Standards

Currently, under various societies, many standards-development projects are ongoing around the world. Global Standards Developing Organizations (SDOs), business groups and partnerships are undertaking these processes, with the latter requiring broad cooperation from the implementation sector and the end-users of these platform-specific standards. Most of these specifications are still being developed, with only a few versions released for review; the vast majority are still in the early blueprint process. It is planned to release further work and preliminary documents of these DLT/Blockchain specifications around 2020. The most important DLT/Blockchain standards currently under review are described below.

1. **IEEE DLT/Blockchain Standards**: The IEEE is developing the IEEE P2418 series, focusing on generic frameworks and architectures, interoperability, building blocks for enabling technology, and vertical industry standards [106,107]. In addition, this system tackles the issues of scalability, security, and privacy in implementation and service [108]. It covers different facets and features of Blockchain, including tokens, intelligent contracts, storing of offchain data, as well as Blockchain that is both permissioned and permission-less.
2. **ISO/TC 307 Blockchain and DLT**: TC 307, driven by the Australian Standards Body and the International Organization for Standardization ( ISO), is an important global initiative. The TC 307 for ISO 307 is in its early stages. Architecture and taxonomy, usage cases, protection and privacy, identification, smart contracts, governance, and interoperability between Blockchain apps are some focal areas here. The first reference design, privacy description, and security of publicly identifiable information will be available by 2021 [109].
3. **Enterprise Ethereum Alliance (EEA)**: This partnership is a member-driven association of standards whose charter is to create transparent Blockchain specifications that facilitate harmonisation and interoperability for global companies and customers [110]. With more than 500 participants working on transparency, standards-based architectures and requirements to speed up the acceptance of Enterprise Ethereum, the EEA is one of the most involved industry partnerships, focused on the creation of software specifications and Ethereum enterprise certification.
4. **ITU-T Blockchain**: In order to examine the standardisation criteria of DLT-based applications and services, the ITU has developed an open participation ITU-T focus group DLT. The key emphasis of the focus group is the recognition and review of DLT-based applications and services, along with the development of best practises and recommendations to facilitate the global deployment of such applications and services [111].
5. **W3C**: The W3C has a Blockchain Community Group working on a Network Ledger Protocol (WLP) to produce ISO 20022-based Blockchain message format standards and create storage usage guidelines, including torrent, public Blockchain, private Blockchain, and side-chain storage [112]. This group will review and analyse emerging Blockchain-related technologies and use cases such as interbank communications, crypto-currencies, etc.

**Figure 5:**
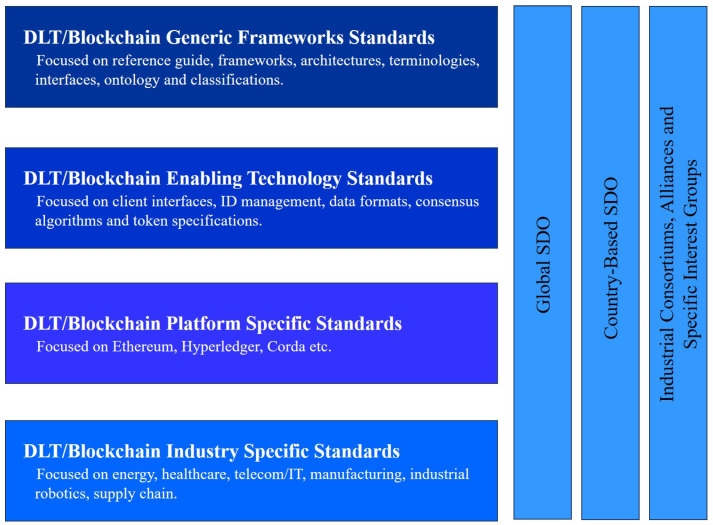
The classification of DLT/Blockchain standards.

Essentially, the above mentioned requirements are commonly categorised as four types of DLT/Blockchain, shown in Figure 5. These divisions are characterised by their points of view, degree of depth, boundaries and demarcation, including industry cooperation with each component of the device and subsystems covered by the technology. It is expected that, in the coming years, there will be an urgent call for cross-chain interoperability among different enterprise-grade (permissioned) and public (permission-less) DLT/Blockchain systems, with various platforms interacting with each other to make the development of DApps much easier and more pervasive [113]. Multiple side-chains (a special type of Blockchain) will be needed and interoperability between these various network segments will be specified using a common-ground protocol, a network agent (e.g., a gateway) or a edge gateway [114]. This would be the ultimate prerequisite for the growth and convergence of DLT technology as a future for decentralised networks and services.

## 4. Physical Unclonable Functions (PUF)

Security and privacy have been a crucial concern in IoE applications in recent years [115]. In fact, IoT Edge technologies have arisen as one of the most serious problems due to electronic devices entering any aspect of smart city applications or energy utilities [116,117]. In addition, due to the trend in globalisation, system integrators have had to cope more than ever with integrated circuit (IC)/intellectual property (IP) counterfeiting. This counterfeit hardware has motivated the need for error-free safe chip authentication, as standard ID or key verification algorithms have been shown to be vulnerable to different forms of cyber attacks [118].

As edge devices become ubiquitous and interconnected in IoE networks must rely on Integrated Circuits (ICs) for data protection and privacy. Providing this protection relies on well-established key generation, data confidentiality and integrity, authentication, and identification [119]. It is therefore necessary for ICs to be able to provide plug-and-play protection in a cost-effective manner. Unfortunately, the classical methodologies [120,121,122] (e.g., digital signatures, encryption, etc.) suffer from numerous weaknesses; they are very sluggish, computationally exhaustive, costly, and increasingly susceptible to physical and side-channel attacks.

Hardware-based security paradigms such as Physical Unclonable Functions (PUFs) [123] and True Random Number Generators (TRNGs) [124] can transcend these limitations and provide random functions on edge modules to create security and reliability for IoE systems. PUFs can be used to retrieve hardware-based chip signatures and eruptive private keys, while TRNGs are used to generate random bits, initialisation vectors and nonces in encryption protocols. It should be remembered that whilst these paradigms will benefit ICs [125,126], there are physical properties and chip architectures that need to be addressed (e.g., power consumption, overhead, heat).

Since IoE and the digitization of energy utilities is a rapidly evolving model, protection and privacy of devices and data are the most challenging criteria for their widespread implementation. Using hardware-based security primitives integrating PUFs [127] and TRNGs [128], it is possible to apply cost-effective security solutions to a large-scale IoT sensor network and even Cyber-Physical Systems (CPSs) sector. Providing a stable environment and forum for IoE systems will defend them from malicious cyber attacks. One of the most critical issues for the production of stable IoT devices is the restricted resource existence of the edge devices. Security historically needs a significant amount of computing capital to perform the computations required for encryption, certificate verification, third-party authentication, etc. By integrating hardware security primitives, i.e., PUFs and TRNGs, developers can easily solve restricted IoT resource reliability and verifiability issues in a cost-effective and efficient manner. In the following sections we shall look into comprehensive details of working and implementation of PUFs for IoE systems [129].

### 4.1. Basics of PUFs

**Figure 6:**
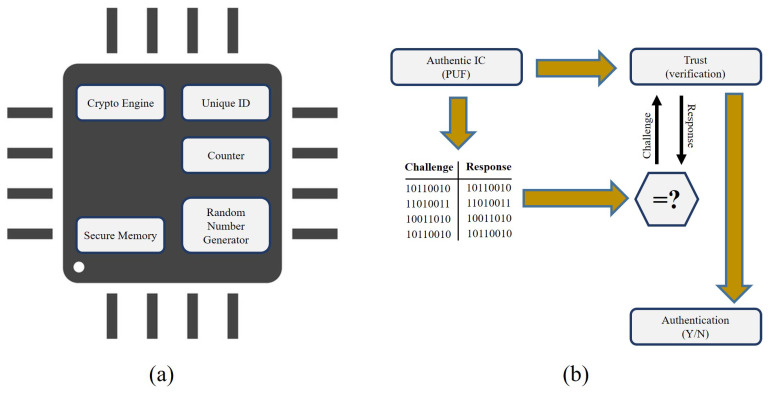
(**a**) Basic design and (**b**) authentication process of Physical Unclonable Function (PUF) chip.

Small differences in the semiconductor manufacturing process make each transistor and each piece of silicon unique. This variants are spontaneous and obtrusive, because it is impossible to create an exact replica of an IC, so we refer to it as Physically Unclonable Function or PUF [130], as depicted in Figure 6a. These differences can be amplified and tested with the normal built-in Static Random-Access Memory (SRAM) cells [131] and the start-up activity of the SRAM chip results in a distinctive pattern that is similar to the IC fingerprint. There are two basic conditions for constructing a PUF: spontaneous and uncontrollable variants. Variations must be random, minimising the probability of producing the same signature [132]. Often, variants must be uncontrollable in such a manner where the adversary cannot replicate the units. The input and output of the PUFs maps a particular set of challenges to the corresponding answer set called Challenge-Response Pairs (CRPs) as shown in Figure 6b. In other words, PUF is a multiple-input (challenges) multiple-output (responses) function that has a dependency on outputs and inputs. The functional relationship between challenge and response is close to that of the random function [133]. Since the PUF is extracted from random process variance, it is very challenging, if not impossible, to predict the answer from a specific problem or to create a function to do so in hardware or software.

#### 4.1.1. Implementation of PUFs

**Figure 7:**
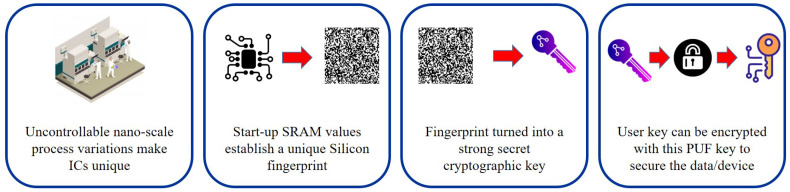
Flow of PUF technology used for secure key management.

PUFs are gradually being used as the root-of-trust hardware for the protection and privacy of IoT devices, data, and services. In order to implement stable applications, the development process variation of the IC can be explicitly used as a source of randomness for the generation of device-unique cryptographic keys, as illustrated in Figure 7. They required minimum on-board power to function as well as memory based on regular SRAM cells (1 kbyte minimum) [134]. In most IoT implementations, edge devices transmit data to the central gateway. It is important to preserve the quality and privacy of these data from the sensors. To preserve the confidentiality of the data to guarantee that it cannot be tampered with, the data can be secured by encrypting the date with the PUF key or mechanisms such as the Message Authentication Code (MAC) and the Advanced Encryption Standard (AES) 256 [135].

The two broad types of application of PUFs are intrinsic and extrinsic [136]. Extrinsic PUFs depend on certain external stimulus to produce CRPs, e.g., light for an optical PUF. Intrinsic PUFs, on the other hand, are those that rely on the inherited internal manufacturing semiconductor properties (process variations) of the system. These implementation methods are summarised in Table 3. The most commonly deployed, cost-effective, and low-energy implementation of PUF is fundamentally the weak SRAM PUF [137,138]. Weak PUFs are basically a modern way to store cryptographic keys on insecure hardware, providing an alternative to read-only memory (ROM), Flash, or other nonvolatile memory (NVMs). Like all PUFs, Weak PUFs have internal, unclonable physical properties, and some sort of challenge-response mechanism that exploits this unusual fingerprint.

**Table 3:** PUFs Taxonomy based on Origin of Stimulus.

PUFs Taxonomy			
Intrinsic			Extrinsic
Memory-Based	Delay-Based	Miscellaneous	Generic
DRAM PUF [139]	Arbiter PUF [140]	Metal Based PUF [141]	Optical PUF [142]
SRAM PUF [138,143]	XOR Arbiter PUF [144]	Photonic PUF [145]	Coating PUF [146]
MRAM PUF [147]	Butterfly PUF [148]	Glitch PUF [149]	
Flash Memory PUF [150]	Bistable Ring PUF [151]	Quantum Confinement PUF [152]	
	Ring Oscillator PUF [153]		

#### 4.1.2. Working of PUFs

Due to differences in the semiconductor manufacturing process, each transistor in an IC has various physical properties as shown in Figure 7. These contribute to minor but observable variations in threshold voltage electronics and gain factors [154]. Although these IC processes’ differences are not completely controllable during development, these physical unit properties cannot be replicated or cloned. Threshold voltages are sensitive to external factors such as temperature and voltage, meaning that their values cannot be used explicitly as special hidden keys or identifiers. Specifically, the activity of the SRAM cell depends on the difference in the threshold voltages of the transistors. Even the slightest variations will be exacerbated and the SRAM cell will be driven into one of the two stable states. Thus, the achieved functional-state is more stable than the underlying threshold voltages, making it the simplest way to use the threshold voltages to create a specific fingerprint [155].

The SRAM memory consists of intertwined SRAM cells. Each SRAM cell consists of two cross-coupled inverters, each consisting of a p- and n-MOS transistor. When the power is added to the SRAM cell, its logical state is determined by the relationship between the threshold voltages of the p-MOS transistors in the inverters. The transistor that begins the first operation decides the result, the logical “0” or “1”. It turns out that every SRAM cell has its own favoured state every time the SRAM is powered due to random variations in threshold voltages. This choice is independent of the preference of the adjacent cells and is independent of the position of the cell on the chip or wafer. Hence an SRAM region yields a unique and random pattern of 0’s and 1’s based on the stimulus applied, as depicted in Figure 6b (i.e., challenges and responses). The long-lasting and reliable efficiency of SRAM PUFs over time makes them the perfect candidate for use in IoE systems. However, the noise characteristics of the SRAM-based PUF reaction have been extensively characterised and evaluated under a wide range of circumstances and semiconductor foundry processes:

- **Temperature**: −55 °C to +150 °C (−67 °F to +300 °F)
- **Voltage Variation**: ± 20%
- **Humidity**: 80%
- **EMC Test**: 3 V/m (EN55020 0.15–150 MHz and IEC 61000-4-3 80–1000 MHz)

**Figure 8:**
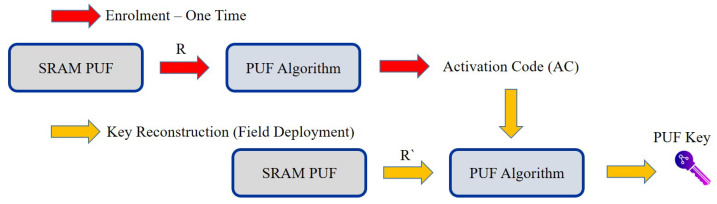
Enrolment and reconstruction phase for the generation of PUF keys. Note that R is the initial PUF response during enrolment, while R’ is a PUF response in the field with a noise component and error correction.

The durability of SRAM PUFs under varied atmospheric conditions has made them the leading candidate for automobile, energy, industrial, and military grade applications. In all the above conditions, the average noise level of the SRAM-based PUF response was found to be less than 15%. Despite this amount of noise, the high-entropy unit can be rebuilt with a special and stable key any time the SRAM is operated [156]. This can be done by applying error correction techniques [157], such as data algorithms and fuzzy extractors, as shown in Figure 8.

### 4.2. PUFs Standardisation

In this section, we will study the state-of-the-art mechanism for standardisation of PUFs. Protection specifications are now being addressed in ISO Sub-Committee ISO/IEC JTC1/SC27 (WG3) as document 20897 [158]. The ISO/IEC DIS 20897 manual defines the protection specifications and test methods for physically unclonable functions for the generation of nonstored cryptographic parameters. The primary reason for this standardisation is to structure and extend the demand for PUFs as solutions for nonmoderable electronic chips identifiers. In addition, this paper further incorporates two redundant protection criteria (i.e., diffuseness and unpredictability) into one (i.e., randomness) which is a stronger output assessment metric for all forms of PUFs. The security requirements for PUFs as mentioned in this document are given here-after:

- **Steadiness**: It is an indicator of the stability of the response time of the PUF. This initiative can be used as a protection necessity. However, PUFs with unsteady responses may be prone to cyber attacks (if the response is very biased) or related key attacks.
- **Randomness**: It assesses the unpredictability of PUF responses when considering the set of stimulus inputs. Ideally, the fingerprints collected could be unpredictable. This protection criterion certifies the unclonability of the PUF.
- **Uniqueness**: It calculates how different the two pairs of different PUFs are. This inter-PUF metric is necessary to measure the degree to which the semiconductor manufacturing process is not capable of producing PUF clones.
- **Unpredictability**: It calculates the difficulty of predicting the response of a (n+1)th PUF that knows all previous “n” instances. This calculation refers to randomness but is more realistic when it includes machine learning or ad hoc research.
- **Unclonability**: This metric means that no easy-to-use bias or parameter occurs by nature in the PUF architecture. The purpose of this protection criterion is to verify the absence of loop holes in PUF systems.

There are various testing parameters that are discussed in the ISO/IEC DIS 20897 document, focusing on the reliability, unpredictability, and diffuseness [159].

- **Reliability**: Bit error rate of 10${}^{-9}$ (or less)
- **Unpredictability**: Entropy of 128 bit (or more)
- **Diffuseness**: Pearson correlation coefficient between challenges and responses of 10${}^{-9}$ (or less)

### 4.3. Cyber Resilience of PUFs

PUFs are gradually being proposed as central building blocks for cryptographic protocols and security architectures for IoT and IoE systems [160]. Unlike other classic cryptographic primitives, where the degree of security can be compared to well-established security proof, the security of PUFs relies on assumptions about physical properties and is a subject of great interest these days [161]. SRAM PUFs can facilitate IoE security by providing integrated, lightweight cryptographic primitives for authentication, and certification without substantial modifications to the design or manufacturing process. However, extreme caution must be taken when developing IoE networks for edge-based PUF devices in order to prevent pitfalls that could endanger protection. SRAM PUFs can facilitate IoE security by providing integrated, lightweight cryptographic primitives for authentication and certification without substantial modifications to the design or manufacturing process. However, extreme caution [162] must be taken when developing IoE networks [163] for edge-based PUF devices in order to prevent pitfalls that could endanger protection.

The most substantial cyber threat to the PUF edge devices are the attackers who can provide the correct response to the challenges [164,165]. It is hard to replicate a PUF because of the semiconductor properties, but with enough computational resources attackers can predict CRPs with a modelling attack [166]. In this review, we are discussing two major attacker models [129]. In the first one, the attacker is able to expropriate the communication framework of devices in the field, whereas, in the second one, an adversary has physical access to the device.

*Man-in-the-Middle (MITM)*

As stated earlier the security of the PUFs is dependent on the size of the CRPs. Large set of CRPs make strong PUFs potentially vulnerable to machine learning assisted cyber attacks only [161]. These attacks originates as MITM and hackers getting hold of the CRPs database of the PUFs. Using this hacked CRPs subset the external hacker tries to process the numerical model to correctly predict the PUFs responses against the challenges given to the device for identification and verification purpose [167]. This attack can further penetrate in the OT field deployed network in IoE, once MITM launches impersonate attack on the neighbouring edge devices and get hold of information on the secret-key generation protocol, i.e., device and data both are under attack. MITM attacks can be performed with comparatively little effort in the IoE, since devices often connect dynamically to the central gateways. An external hacker can place a low-cost computing device in proximity to the attacked device and let it join the same (possibly encrypted) wireless network. Therefore, the risk for such an attack is relatively high, and suitable defense mechanisms [42] must be used, i.e., lightweight cryptography or Blockchain for the verification a identification of the devices.

*Side Channel Attacks*

This type of cyber attack originates when the adversary physically get access to the device. They can be classified as invasive, semi-invasive, and noninvasive attacks [168]. Invasive attacks are the type of cyber threat where the hacker gets hold of the physical device and accesses the internal circuitry. Though it is not very common, as any physical access can damage the PUF chip but there are reports that prove that PUFs are in fact vulnerable against invasive attacks, including creating a full physical clone of a PUF [169]. Usually these attacks need proper knowledge of input/output interfaces, communication protocols, and penetration testing skills. Above all, physical access to the device made these attacks less attractive in modern smart grid applications. Moreover, physical cloning needs the replication of time delay, synchronisation, and error correction modules along with the PUF chip, making it hard to achieve the right combination to get exact CRPs.

Timing attacks, power monitoring attacks, electromagnetic attacks and differential fault detection are prominent attacks in this group [170]. Timing attacks generally require mathematical analysis of the timing required by a CPU to carry out cryptographic operations and thereby decide the secret key. However, instead of secret keys, PUFs use a challenge response system and it is considerably more difficult to calculate the timing delays of a circuit in an IC, reliably. Power monitoring attacks depend on monitoring power usage during computations. We may, however, make PUFs stable against these attacks by designing the PUF in such a way that the number of zeros and ones in the latches is constant. Whereas, in electromagnetic attacks, PUFs are exploited by inserting faults into protection hardware by exposing it to irregular environmental conditions, as differential fault analysis is done. Although PUFs are pretty sensitive to the environmental conditions, these attacks can be mitigated by opting for a delay-based modification in the PUF design [171].

A few other prominent attack [128] types in PUF devices are:

- **Overbuilding**: These types of cyber attacks originate because of the lack of intellectual property (IP) on the chip information or devices. The extra devices can be then sold in open market. The design could also be sold to third parties and PUF devices can be replicated easily.
- **Theft-of-Service**: In the era of digitisation, the edge devices are virtualised with different services and processing algorithms from the cloud. In case of any adversary on the IoE nodes, the services not authorised to some of the nodes can be accessed by creating a perfect clone or CRPs.
- **Denial-of-Service**: The regular upgradation of the edge devices in IoE should be audited and encrypted. For any malicious node, if it can get the respective upgraded firmware code it can launch the cyber attacks. This results in the malfunctioning of the operations and denial-of-service attack. The PUF developer should design firmware update procedures very securely to make sure they cannot be used without proper authentication.

The PUF modules can be modified to mitigate the external cyber attacks. One of the well-known solutions is Controlled Physical Unclonable Functions (C-PUFs) [44]. The C-PUF is accomplished in this scheme using several layers. For example, by avoiding a chosen challenge attack on PUF, one random hash function before the PUF can be modelled to avoid the MITM attack. This strategy does not allow the hacker to extract the PUF parameters using the model-based adversary attack. In order to have another layer of security, an error correcting code is placed after the PUF output to reduce the noisy output measurements which result in more resilient responses. The second solution for mitigating the cyber attack is via Public Physical Unclonable Functions (P-PUFs) [172]. This methodology is used to prevent the side-channel attacks by delaying the response from the gates (XOR).

The IoE network designers can have end-to-end system security by opting the hybrid software-hardware approach. Software encryption is deemed to be cost-effective and relatively easy to implement and update [173]. However, software security is relatively easy to modify, and malware can infiltrate or penetrate the software. Hardware-based security is known to be a more powerful choice for IoE. Hardware protection makes it impossible for the OT networks to breach [174] because it is hard to adjust the physical layer and protect ICs with the root of confidence. CRPs should be encrypted and installed in the safe ROM of the microcontroller, the root of confidence includes trustworthy functionality that can be used to validate and authenticate the [175] software signature of the program. By introducing a hardware-based root-of-trust, designers will basically close more possible entry points into their work.

## 5. Proof-of-PUF Authentication in IoE

Data management, security and privacy of data, devices, and individual SCADA systems are some of the key aspects in the IoE architecture that need to be resolved. Integrating the Blockchain into the IoE environment can help solve these issues and helps in achieving a unified secure data technology [176]. In this section, we will review the implementation and the performance of a hybrid framework of Blockchain, based on the secret computational model of a PUF [177,178,179,180], also known as PUFChain. The key factors that make this algorithm a leading candidate for concurrent data and device security in an IoE network are:

- Key storage and key vault in high-security and mass-volume edge devices
- Secure cloud integration (OT/IT Convergence)
- Preventing reverse engineering of IP and software
- Combating counterfeiting and cloned devices
- Securing FPGAs and ASICs

In an IoE environment, most of the devices are low power and low computational devices. Various other characteristics of the devices, such as security and privacy, power consumption, on-board memory, and ability to host computational heavy algorithms [181] become bottlenecks when integrating a Blockchain environment into the IoE. The Blockchain has been computationally intensive especially in a dense node network. So there are some challenges that need to be dealt with before integrating it into IoE sector [182]. To overcome this limitation, PUFChain has been proposed [177,178,183] for resource constraint devices. The combination of PUF and hash decreases the numerical complexity of the processing, making it suitable for integration into most scenarios. With the PUF’s ultra-low power architectures, power overhead can also be drastically minimised to achieve low-carbon networks. In addition, Proof-of-PUF authentication is known to be a lightweight consensus algorithm that can easily guarantee protection and privacy in a scalable manner [184]. Initial findings [177] on PUF-based authentication showed 1000x faster processing times than well-established PoW and 5x faster than previously recorded hardware-based solutions. Transaction time reduction relative to hardware dependent implementations is 79.15%. This is due to the fact that PUFChain uses a hybrid PUF and hash system that does all the requisite cryptographic processing. The mining method is then offloaded to the hardware module, which reduces loading times.

### 5.1. Hardware Assisted Proof-of-PUF Authentication

**Figure 9:**
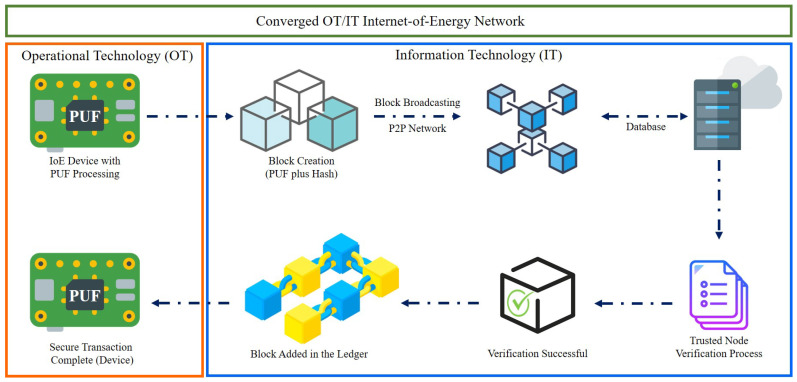
Networking architecture of PUFChain.

The basic network of the PUFChain architecture is seen in Figure 9. IoE systems deployed in the sector, also known as Operational Technology (OT), track large quantities of critical energy infrastructure data. The signature PUF and hash is applied to the system at the OT stage of the PUFChain architecture. This lowers the computing pressure on the edge gateways, decreases the bandwidth, and increases the latency requirements of the stable IoE network [185]. A variety of transactions are initiated, validated, and linked to the Blockchain for authentication and identification purposes. A two-step protocol was proposed where, after the implementation of a new IoE device into the network [186], the device needs to go through the enrolment phase.

The nodes in the PUFChain architecture, as in Figure 9, can be categorised into two main types; (a) client nodes, that are responsible for data collection and broadcasting it in the network and (b) trusted nodes, that are assigned the task to authenticate the end-node devices and broadcast it back to the network. There are multiple trusted nodes available in the Blockchain network that can extract the device ID, PUF challenge input, and the hash from the block data coming from the clients. The trusted node obtains the response outputs from the encrypted database by using the device ID. At the trusted node, the extracted response, system ID, and sensor data from the block are sent to the hashing module and a hash is determined. The node is authenticated and the block is broadcasted to the network for the rest of the devices to connect it to their local Blockchains, if the hash in the block and the created hash correlate with each other. If the hash does not match, the procedure is replicated with the device ID for the rest of the keys present in the encrypted database. The block is discarded if none of the hashes correlate.

PUFChain guarantees safe OT/IT integration in IoE networks with reduced complexity and also provides concurrent device/data protection. Centred on the Proof-of-PUF authentication, Blockchain stores data that has been encrypted with identical PUF-derived keys and attributes, offering immutable confirmation that data has not been tampered with, in addition to providing traceability and straightforward auditing capabilities [187]. The protection of the PUFChain is dependent on the randomness of the PUF technology as discussed in the preceding sections. For PUF responses to be more stable and immune to foreign cyber threats, the number of 0s and 1s should be the same. For example, there should be 64 0-bits and 64 1-bits in the output key of 128 bits. This makes the PUF response more stable and immune to numerous attacks by [177]. PUFs together with Blockchain are exciting cryptographic primitives. On the other hand, the commercial problems for the large-scale implementation of this strategy in IoE networks are the probability of the randomness and stability properties of PUFs. We should strive to mitigate risk factors and develop effective error reducing strategies for PUFChain.

### 5.2. Enrolment and Authentication Steps in Proof-of-PUF Authentication

**Figure 10:**
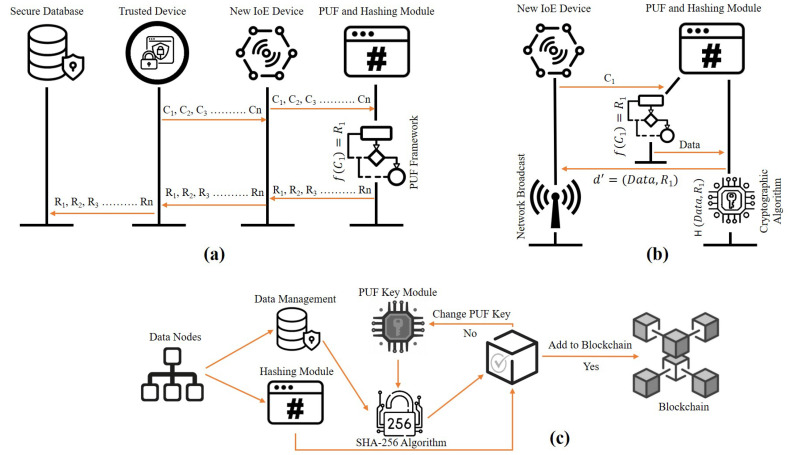
(**a**) Device enrolment steps, (**b**) Transactions initiation steps and (**c**) Device authentication steps in Proof-of-PUF authentication [177].

This section presents the enrolment and authentication steps of PUFChain based devices [187]. The use-case under consideration in this review paper is a resource rich edge-gateway communicates with constrained IoE devices in an untrusted field. The two basic assumptions regarding this untrusted sector are that, since there is so little or no control over the devices, device security is vulnerable to both passive and active cyber attacks. The different steps involved in various phases of the Proof-of-PuF authentication are presented in Figure 10. The first step, as explained in Figure 10a, is the device enrolment phase. IoE usually have a dense operational network (i.e., 100s of devices). Those end-nodes need to be enrolled and authenticated before they act as a client. There is a PUF module on every end-node device in the network that can produce unique identification numbers that are later used in the enrolment process for each device. The secure database is linked to the multiple trusted nodes in the network. A collection of challenge inputs ((*R*) and (*C*) functions) for the PUF module will be chosen during the enrolment stage [188]. These challenge response pairs are stored in the secure database that can be accessed by the trusted nodes of the IoE network.

If the edge node is securely enrolled in the network, it becomes capable of initiating active transactions authenticated by a trusted server, as depicted in Figure 10b. The edge devices in the IoE network collect data from the sensors and initiate the transactions. In this case, the transaction data includes the sensor data obtained and the device’s MAC address. For the rest of the nodes in the network and the trustworthy node, the MAC address acts as the identifier. It is sent to the hardware accelerator until the transaction is produced. It includes the PUF and the cryptographic hash can be computed. Before it is connected to the Blockchain, the block that is broadcast to the network needs to be approved by the trusted node. Once the block is received by the trusted node, the data (D${}_{n}$) and the hash (H${}_{n}$) are retrieved, Figure 10c. These two parameters help the IoE network to verify and authorise the edge nodes. In addition to the special identity that the PUF produces, the MAC address is used. The trustworthy node gets the PUF keys from the secure database with the help of the MAC address. To execute the cryptographic hash, the PUF key and the data are sent to the hardware accelerator. The input data and the PUF key are hashed and the denouement hash is compared to the H${}_{n}$ received from the node. If both the hashes match, the device is authenticated. If they do not match, the process continues for all the PUF keys that are stored in the database during the enrolment phase of the device. The associated algorithms for all the three phases, as in Figure 10a–c, are explained in [177].

### 5.3. Hardware Implementation of PUFChain

**Figure 11:**
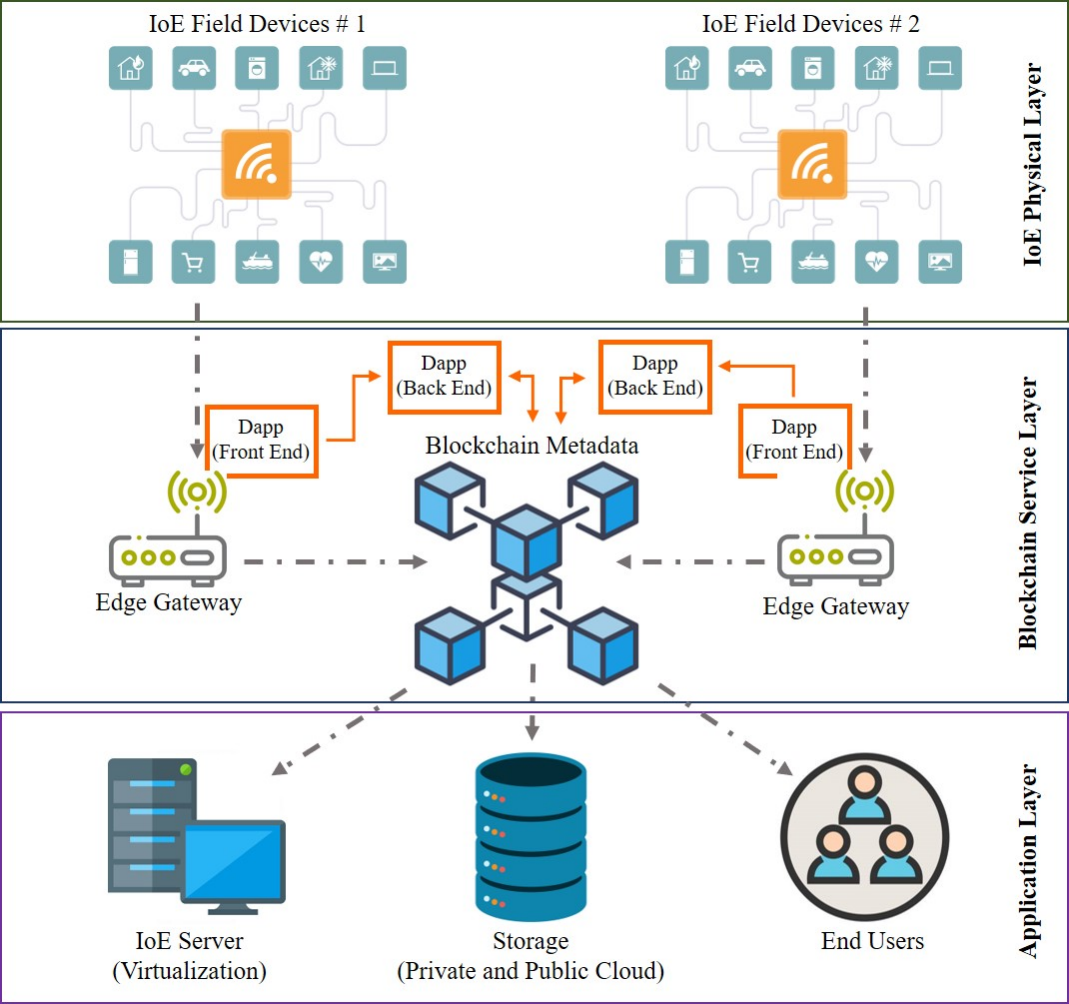
Proof-of-Concept Layered Architecture for IoE-Blockchain Hardware Integration.

Developments in electronics and wireless networking technology have led to exponential advances in a stable, mobile, and smart environment. This has resulted in an growth in the number of appropriate mobile devices in many countries, a decrease in their cost of manufacture, and a paradigm change from the physical world to the digital world. Due to the density of IoE edge devices in the region, energy utilities opt for low-cost but effective devices that can provide smooth integration [189]. Classical IoT systems are extremely centralised architectures that suffer from a range of technological shortcomings, such as cyber threats and single failure points. To overcome this bottleneck, IoT networks can quickly follow a decentralised Blockchain-PUF model while retaining real-time monitoring and control between end-user and edge gateways. Blockchain and PUF can complement IoE by providing a secure sharing service where information is reliable and traceable. Data sources may be detected at any time and data stays immutable over time, thereby increasing its security [190]. This section summarises the hardware implementation of Blockchain and PUF based IoT networks, as depicted in Figure 11, using the cost effective devices, i.e., Arduino, Raspberry, and Orange Pi boards.

**Figure 12:**
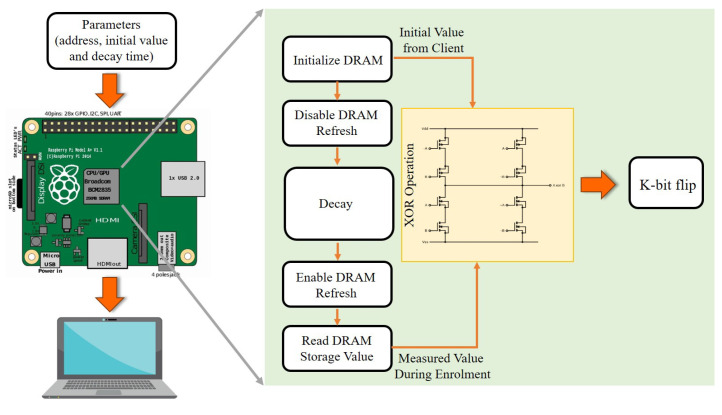
Structure of DRAM PUF implementation on Raspberry Pi.

For the cost-effective deployment of the Blockchain-based IoE network, a layered approach has been implemented to simplify the hardware specifications of [191]. With advances in hardware technologies, edge devices such as Raspberry Pi (RPi) will directly benefit from network resources by invoking state-of-the-art Representation Transition Application Programming Interfaces (REST APIs). This layered, focused approach, as seen in Figure 11 allows the IoE network maximum versatility for potential expansions and developers can substitute or install any new module without disrupting the rest of the [73,189] infrastructure. However, it is important to know that not all forms of PUFs can be introduced by using cost-effective hardware tools such as RPi. Decay-based DRAM-PUF [192] is the most common technique that can be used in IoE networks. The implementation design of the RPi is seen in Figure 12, with three key parameters: PUF address, initial value, and decay time. The address of the DRAM may be used as a PUF identifier. The initial value is the set of digital data that was used before the PUF query to trigger the PUF identifier. In the PUF query, decay-time determine how long the DRAM PUF has been disabled. These parameters are then compiled with the aid of the RPi Universal Asynchronous Receiver/Transmitter (UART). Then the PUF query code running on the Graphics Processing Unit (GPU) can retrieve these Central Processing Unit (CPU) parameters from the mailbox. The programs running on the CPU and GPU can only communicate through mailboxes [192].

Researchers are still designing mainstream platforms for the deployment of Blockchain in IoE [190], but few of them have earned positive consideration from the consortium, i.e., Ethereum [193] and Hyperledger Fabric [194]. The summarised comparison of the Blockchain platforms for creating IoE applications are given in the Table 4.

To test the viability of running Blockchain-PUF platforms on IoE devices, the execution time to register the devices, sensor readings, data query, and block processing overheads have been extensively analysed in [191] and are enlisted in Table 5. Blockchain based IoE nodes can be implemented via Raspberry Pi3 [195] with 1GB on-board memory, Android Things v0.8 operating system, CoAP server, Android Studio for integrated development environment, and programming language support such as Java, Python etc. Connection with the centralised system and the IoE nodes usually uses the restricted application protocol (CoAP) while HTTP is used for communication between the system host and the Blockchain network. It is critical that the architecture of hardware and network infrastructure should be robust to significant network traffic. Although the hybrid Blockchain-PUF and IoE research studies are still in its infancy, this review article explores the potential applications of IoE and Blockchain to improve efficiency and bring automation, to revolutionise robust business solutions in energy sector. Current and future research challenges are discussed in the later section, aiming at testing the interoperability of the cost-effective system with different IoE frameworks.

## 6. Applications and Use-Cases

The most common applications of PUFs proposed in the relevant literature [43,136] include identification, authentication, attestation, secure boot, anticounterfeiting, and secure key agreement protocols. Additionally, PUFs can serve as the basis for the implementation of true ultra-high entropy random number generators. PUFs can also be combined with other security primitives and entropy sources for combine data and device security, so that the overall architecture will produce a divergent unique response for the same challenge, when queried at different times. Since edge devices and PUFs are cost-effective monitoring solutions for IoE [196], they can be incorporated in the network for the following applications.

- **Authentication**: PUFs on devices can be used for authentication and binding hardware to software platforms, secure key storage, key-less secure communication in the OT/IT convergence. Two different schemes of authentication exist, namely client authentication and server authentication, both being based on the CRPs.
- **Device Identification**: The use of PUFs for device identification purposes effectively turns the device into the authentication token. It reduces the necessity to store cryptographic keys and look-up tables inside the device, which can be a cyber threat and adversary can extract cryptographic material from nonvolatile memory. Instead, the PUF device generates an transient key “on-the-fly” on the basis of it‘s unique fingerprint, minimising the attack surface to extract the key.
- **Random Number Generation**: An important building block for many cryptographic systems is the random number generator. Random numbers with high entropy are required in these frameworks, because they are unclonable for potential attackers. Since an interesting source of randomness is readily available in PUFs due to semiconductor manufacturing process. This property can be exploited to use PUF as a truly random number generator.
- **Secure Environment**: The idea of this application is to provide hybrid software-hardware security and generate a cryptographic key. Subsequently, the key is used to decrypt software, which is installed on the chip. The basic function is to decrypt the boot-loader, which is executed first during PUF start-up or embedded devices. After the boot-loader has been decrypted using the key, derived from the PUF response, it subsequently unlocks the kernel, which in turn decrypts user space applications.
- **IP Protection**: The integrated circuit (IC) design flow is globalised due to increase in design, fabrication, testing, and verification costs. While globalisation has provided cost benefits and reduced the time-to-market, it has introduced several attacks such as piracy, malicious modifications, and counterfeiting. IP of the devices can make use of unique PUFs fingerprint to protect the devices.

### IC Traceability via PUF and Blockchain (Use-Case)

Driven by the continuous and exponential scaling of semiconductor manufacturing technologies, integrated circuits (ICs) and their installation in the IoE have been more complex than ever before [197]. The globalisation of the IC supply chain has raised the possibility of counterfeit, tampered, and repackaged chips on the market with the least protection from IP. Protection of device updates, firmware, and contact networks has gained a lot of attention due to various hardware bugs, threats, and cyber attacks. The security aspect of ICs and electronic devices has been restricted to a range of bugs and attacks, such as side-channel analysis that exploits hardware implementation of cryptographic algorithms for leaking hidden keys and invasive/semi-invasive attacks, as discussed in the previous section. However, the credibility of the supply chain of ICs and electronic systems is equally critical, as hardware derived from an untrusted supply chain cannot act as the underlying root-of-trust hardware [198].

**Figure 13:**
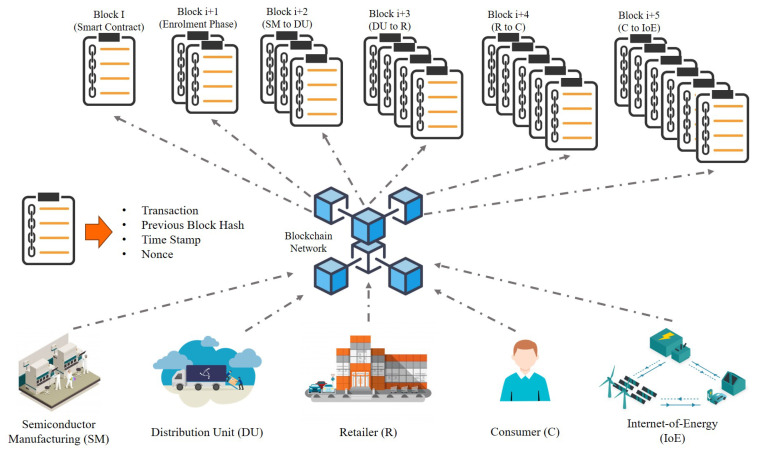
IC traceability from manufacturer to the end-user via PUF and Blockchain.

With the new developments in PUF and Blockchain [176], an end-to-end protected IC traceability can be accomplished, thanks to Blockchain, a public immutable ledger that holds an ever-increasing collection of data records (ICs, PUFs, and CRPs) guarded from tampering and revision, as seen in Figure 13. The authenticity of IP knowledge and traceability is a critical case-study for IoE, which refers to the combination of the ability to know the actual possession of the edge system at all times (track) and the ability to find the origin [199], the history of ownership, the time spent at each point (trace) by means of registered identifications via Blockchain. The supply chain of semiconductor products, from processing to installation of OT devices and product-life, is protected by blocks containing PUF specific signatures, hash and time stamps, transactions, never forming a chronological chain, as seen in Figure 13. It can make it possible to validate, record, and deter any malicious party from modifying or questioning the authenticity of the information reported by IP authentication and IC proprietary transmission of information [200]. This verifiable and permanent ledger will also allow the identity and traceability of the IC across the entire supply chain and its lifetime of deployment. Real-time monitoring of IP secured devices across several supply chains includes a clear, tamper-proof meta-data network architecture [201], i.e., edge computing and cloud convergence, which is not only trusted by all stakeholders but also adaptable to changing markets and regulations.

## 7. Current Challenges and Future Research

The promising and commercial implementation of hybrid approach, i.e., Blockchain and PUF, is leading the security of smart grid applications. Despite the wide spread use in IoE systems, there are significant challenges in the development and deployment of existing and planned systems that will need further investigation:

- **Blockchain Design Types**: Decision from the energy utilities of whether your Blockchain design will be a public Blockchain (generally open to participation by anyone and not permissioned), private Blockchain (involving limited participation and having permission structures), or a hybrid (Blockchain systems with both public and private designs). Specially, privacy laws and data jurisdiction/sovereignty should be considered for all of the digital data on the system.
- **Scalability**: One major technology challenge of Blockchain and PUF is related to the technical scalability and integration of the network which can put a strain on the adoption process, especially for public Blockchains. As more and more edge devices are integrated into the legacy energy networks, they need to be verified, authenticated, and orchestrated with existing Blockchain network.
- **Standardisation and Interoperability**: Another vital challenge is the lack of interoperability between the large number of Blockchain networks that coexist in the same smart grid domain. The lack of such uniformity across Blockchain frameworks also takes away consistency from basic processes like security, making network wide adoption an almost impracticable task.
- **Seamless Integration with IoE**: This is the biggest challenge for energy utilities and IoE systems to integrate the advancements with legacy systems. In most cases, if they decide to use Blockchain and PUF, the organisations are required to completely restructure their classical system or design a way to successfully integrate the two technologies.
- **Data Sharing and Access**: Sharing and access to critical monitoring data is subject to various privacy, legal, and regulatory laws. Decisions need to be made about what type of data will be shared with the Blockchain participants. Thoughtful network architecture is required to see if data will be stored on-chain, offchain or on a side-chain, and the type of permission structures that will be utilised.
- **Productivity Paradox**: The effectiveness with which PUF and Blockchain networks can execute peer-to-peer data transfer comes at a high aggregate cost/bandwidth, which is greater for a dense edge system. This inefficiency come into play because each node performs the same tasks as every other node on it’s own copy of the data in an attempt to be the first to find a solution.

## 8. Conclusions

To comprehend the secure IoE development, this review paper provides the current state-of-the-art on the Blockchain technology and PUFs by employing Proof-of-PUF as a consensus algorithm (PUFChain). Devices constituting the IoE have become widely used in energy utilities, therein generating a large amount of data from critical infrastructure that needs an end-to-end security and privacy solution. The pragmatic industrial option is PUFchain, which is an efficient security framework that can have a seamless integration in an IoE network inherently having energy and processing power constraints. Classical authentication mechanisms can no longer be based on simply storing secret data or PKI as the secret keys on the edge devices that could get stolen or leaked. This issue is addressed in PUFchain where physical properties of the devices are used as unique fingerprint and recorded as a proof of authentication. The added benefit of PUFChain for the IoE networks is that PUF key generated is not transmitted over the communication channel in the Proof-of-PUF consensus algorithm which not only makes the system resilient to communication attacks but also eases the bandwidth and latency requirements.

## Figures and Tables

**Table 4:** Blockchain methodologies for designing IoE applications.

Platform	Blockchain	Consensus	Smart Contracts
Ethereum	Public and Permission-Based	PoS	Yes
IBM Watson Hyper-ledger	Permission-Based	PBTF/SIEVE	Yes
Multichain	Permission-Based	PBTF	Yes
Litecoin	Public	Scrypt	No
Waltonchain	Permission-Based	PoC	No
Lisk	Public and Permission-Based	DPoS	Yes
Edenchain	Permission-Based	PoS	Yes
Quorum	Permission-Based	Multiple	Yes
Moeco	Private and Permission-Based	Exonum	Yes
HDAC	Permission-Based	ePoW, Trust-based	Yes
IOTA	Permission-Based	PoW	Yes
NetObjex	Permissionless	Hedera Hashgraph	Yes

**Table 5:** Performance Analysis of Blockchain-based IoE Hardware.

	Service Execution Time (ms)					
	Device Registration			Sensor Reading		
	Min	Avg	Max	Min	Avg	Max
**50 Devices**	2262	2286	2375	1974	2490	2770
**150 Devices**	2257	2335	2801	1696	2679	3005
**250 Devices**	2254	2585	3004	2043	2767	3322
**500 Devices**	2267	2923	4013	2689	3012	4983

## Abbreviations

The following abbreviations are used in this manuscript:

5G	Fifth Generation Cellular Network
AES	Advanced Encryption Standard
ASICS	Application-Specific Integrated Circuits
AI	Artificial Intelligence
BaaS	Blockchain-as-a-Service
CAGR	Compound Annual Growth Rate
CAPEX	Capital Expenses
CPS	Cyber Physical System
CRPS	Challenge-Response Pairs
CS	Cybersecurity
DAppss	Dated, Achievable, Personal, Positive and Specific
DLT	Distributed Ledger Technology
DNOs	Distribution Network Operators
DPoS	Delegated Proof-of-Stake
DTs	Digital Twins
DUT	Device Under Test
E2E	End-to-End
EEA	Enterprise Ethereum Alliance
EMC	Electromagnetic Compatibility
FIB	Focused Ion Beam
FPGAs	Field Programmable Gate Arrays
ICs	Integrated Circuits
ICT	Information and Communication Technology
IoE	Internet-of-Energy
IIoT	Industrial Internet-of-Things
IoT	Internet-of-Things
IP	Intellectual Property
ISO	International Organization for Standardization
IT	Informational Technology
ITU-T	International Telecommunication Union
LTE	Long Term Evaluation
LPWAN	Low-Power Wide-Area Network
MAC	Message Authentication Code
MITM	Man-in-the-Middle
NVM	Non-Volatile Memories
OPEX	Operational Expenses
OT	Operational Technology
PBFT	Practical Byzantine Fault Tolerance
PKI	Public Key Infrastructure
PoP	Proof-of-Physical Unclonable Function
PoS	Proof-of-Stake
PoW	Proof-of-Work
PUF	Physical Unclonable Function
PUFChain	Physical Unclonable Function assisted Blockchain
REST	Representational State Transfer
ROM	Read-Only-Memory
SCADA	Supervisory Control and Data Acquisition
SDOs	Standards Developing Organization
SRAM	Static Random-Access Memory
TRNGs	True Random Number Generators
WLP	Web Ledger Protocol
